# Supplementary material for: Machine Learning Accelerates Crystallization for Structure Determination
Source: Angew Chem Int Ed Engl. 2026 May 4;65(25):e1218503. doi: 10.1002/anie.1218503 (PMC13266958; doi:10.1002/anie.1218503)

checkCIF/PLATON report

Structure factors have been supplied for datablock(s) lcz064

THIS REPORT IS FOR GUIDANCE ONLY. IF USED AS PART OF A REVIEW PROCEDURE FOR PUBLICATION, IT SHOULD NOT REPLACE THE EXPERTISE OF AN EXPERIENCED CRYSTALLOGRAPHIC REFEREE.

No syntax errors found. CIF dictionary Interpreting this report

Datablock: lcz064

Bond precision:	C-C = 0.0232 A		Wavelength=1.54184
Cell:	a=13.2452(2)	b=11.4403(2)	c=22.7510(3)
	alpha=90	beta=90.498(1)	gamma=90
Temperature:	100 K		

	Calculated	Reported
Volume	3447.31(9)	3447.31(9)
Space group	P 21	P 1 21 1
Hall group	P 2yb	P 2yb
Moiety formula	C29 H11 Ag3 F18 N6 O3	2(C15 H3 Ag3 F18 N6), 2(C14 H8 O3)
Sum formula	C29 H11 Ag3 F18 N6 O3	C58 H22 Ag6 F36 N12 O6
Mr	1157.05	2314.09
Dx,g cm-3	2.229	2.229
Z	4	2
Mu (mm-1)	14.867	14.867
F000	2216.0	2216.0
F000’	2226.68	
h,k,lmax	16,14,28	16,14,28
Nref	14757[7759]	12237
Tmin,Tmax	0.131,0.125	0.509,1.000
Tmin’	0.036	

Correction method= # Reported T Limits: Tmin=0.509 Tmax=1.000
AbsCorr = MULTI-SCAN

Data completeness= 1.58/0.83 Theta(max)= 77.931

R(reflections)= 0.0567(11102) wR2(reflections)= 0.1442(12237)

S = 1.027 Npar= 1060

The following ALERTS were generated. Each ALERT has the format
test-name_ALERT_alert-type_alert-level.
Click on the hyperlinks for more details of the test.

Alert level B

```
PLAT342_ALERT_3_B Low Bond Precision on  C-C Bonds ..............    0.02321 Ang.
```

Author Response: Disordered structure.

```
PLAT780_ALERT_1_B Coordinates do not Form a Properly Connected Set     Please Do !
```

Author Response: The alert is due to a large number of co-crystallized molecules in the which sometimes do not show as a connected set. This is acceptable from a crystallographic point of view.

Alert level C

```
STRVA01_ALERT_4_C           Flack test results are ambiguous.
           From the CIF: _refine_ls_abs_structure_Flack    0.470
           From the CIF: _refine_ls_abs_structure_Flack_su    0.015
PLAT041_ALERT_1_C Calc. and Reported SumFormula    Strings  Differ     Please Check
              Calc: C29 H11 Ag3 F18 N6 O3
              Rep.: C58 H22 Ag6 F36 N12 O6
PLAT042_ALERT_1_C Calc. and Reported MoietyFormula Strings  Differ     Please Check
              Calc: C29 H11 Ag3 F18 N6 O3
              Rep.: 2(C15 H3 Ag3 F18 N6), 2(C14 H8 O3)
PLAT090_ALERT_3_C Poor Data / Parameter Ratio (Zmax > 18) ........       7.09 Note
PLAT213_ALERT_2_C Atom F4               has ADP max/min Ratio .....       3.6 prolat
PLAT213_ALERT_2_C Atom F015             has ADP max/min Ratio .....       3.1 prolat
PLAT213_ALERT_2_C Atom F017             has ADP max/min Ratio .....       4.0 prolat
PLAT213_ALERT_2_C Atom F013             has ADP max/min Ratio .....       3.9 prolat
PLAT213_ALERT_2_C Atom F02T             has ADP max/min Ratio .....       3.1 prolat
PLAT220_ALERT_2_C NonSolvent   Resd 1  F   Ueq(max)/Ueq(min)  Range       3.2 Ratio
PLAT230_ALERT_2_C Hirshfeld Test Diff for    F03A     --C5        .       6.5 s.u.
PLAT234_ALERT_4_C Large Hirshfeld Difference F031     --C022      .      0.23 Ang.
PLAT234_ALERT_4_C Large Hirshfeld Difference F034     --C027      .      0.19 Ang.
PLAT234_ALERT_4_C Large Hirshfeld Difference O1       --C01S      .      0.20 Ang.
PLAT234_ALERT_4_C Large Hirshfeld Difference N00Z     --C023      .      0.17 Ang.
PLAT234_ALERT_4_C Large Hirshfeld Difference F012     --C02N      .      0.19 Ang.
PLAT241_ALERT_2_C High   'MainMol' Ueq as Compared to Neighbors of       O00U Check
PLAT242_ALERT_2_C Low    'MainMol' Ueq as Compared to Neighbors of       C01S Check
PLAT369_ALERT_2_C Long   C(sp2)-C(sp2) Bond  C01X     - C02E      .      1.54 Ang.
PLAT911_ALERT_3_C Missing FCF Refl Between Thmin & STh/L=    0.600          7 Report
```

```
               0   2   0, -11   9   4, -12   4 12, -14   0 13, -10   7 16, -10   0 17,
              -2   0 27,
PLAT915_ALERT_3_C No Flack x Check Done: Low Friedel Pair Coverage         68 %
PLAT971_ALERT_2_C Check Calcd Resid. Dens.  1.35Ang From C02S            1.88 eA-3
PLAT971_ALERT_2_C Check Calcd Resid. Dens.  1.44Ang From C5              1.73 eA-3
PLAT977_ALERT_2_C Check Negative Difference Density on H1       .       -0.51 eA-3
```

Alert level G

```
PLAT003_ALERT_2_G Number of Uiso or U(i,j)  Restrained non-H-Atoms         25 Report
PLAT004_ALERT_5_G Polymeric Structure Found with Maximum Dimension          1 Info
PLAT007_ALERT_5_G Number of Unrefined Donor-H Atoms .............           2 Report
              H1    H026
PLAT033_ALERT_4_G Flack x Value Deviates > 3.0 * Sigma from Zero .      0.470 Note
PLAT045_ALERT_1_G Calculated and Reported Z Differ by a Factor ...          2 Check
PLAT083_ALERT_2_G SHELXL Second Parameter in WGHT  Unusually Large      34.98 Why ?
PLAT171_ALERT_4_G The CIF-Embedded .res File Contains EADP Records          1 Report
PLAT177_ALERT_4_G The CIF-Embedded .res File Contains DELU Records          1 Report
PLAT178_ALERT_4_G The CIF-Embedded .res File Contains SIMU Records          2 Report
PLAT186_ALERT_4_G The CIF-Embedded .res File Contains ISOR Records          3 Report
PLAT188_ALERT_3_G A Non-default SIMU Restraint Value has been used     0.0100 Report
PLAT188_ALERT_3_G A Non-default SIMU Restraint Value has been used     0.0100 Report
PLAT192_ALERT_3_G A Non-default DELU Restraint Value for SecondPar     0.0200 Report
PLAT242_ALERT_2_G Low    'MainMol' Ueq as Compared to Neighbors of         C5 Check
PLAT242_ALERT_2_G Low    'MainMol' Ueq as Compared to Neighbors of       C01L Check
PLAT242_ALERT_2_G Low    'MainMol' Ueq as Compared to Neighbors of       C022 Check
PLAT242_ALERT_2_G Low    'MainMol' Ueq as Compared to Neighbors of       C027 Check
PLAT242_ALERT_2_G Low    'MainMol' Ueq as Compared to Neighbors of       C02A Check
PLAT242_ALERT_2_G Low    'MainMol' Ueq as Compared to Neighbors of       C038 Check
PLAT242_ALERT_2_G Low    'MainMol' Ueq as Compared to Neighbors of       C016 Check
PLAT242_ALERT_2_G Low    'MainMol' Ueq as Compared to Neighbors of       C01C Check
PLAT242_ALERT_2_G Low    'MainMol' Ueq as Compared to Neighbors of       C01T Check
PLAT242_ALERT_2_G Low    'MainMol' Ueq as Compared to Neighbors of       C020 Check
PLAT242_ALERT_2_G Low    'MainMol' Ueq as Compared to Neighbors of       C02N Check
PLAT242_ALERT_2_G Low    'MainMol' Ueq as Compared to Neighbors of       C02R Check
PLAT333_ALERT_2_G Large Aver C6-Ring C-C Dist C00M     -C01X     .       1.46 Ang.
PLAT333_ALERT_2_G Large Aver C6-Ring C-C Dist C00W     -C02M     .       1.45 Ang.
PLAT434_ALERT_2_G Short Inter HL..HL Contact F4       ..F00X     .       2.77 Ang.
                                                    x,y,z  =     1_555 Check
PLAT720_ALERT_4_G Number of Unusual/Non-Standard Labels ..........        136 Note
              Ag01    Ag02    Ag03    Ag04    Ag05    Ag06    F007    F008
              F009    F00A    N00B    O00C    F00D    N00E    F00F    N00G
              F00H    O00I    N00J    F00K    F00L    C00M    F00N    F00O
              N00P    F00Q    F00R    N00S    N00T    O00U    N00V    C00W
              F00X    F00Y    N00Z    N010    F011    F012    F013    F014
              F015    C016    F017    C018    N019    N01A    C01B    H01B
              C01C    F01D    F01E    C01F    H01F    C01G    O01H    C01I
              H01I    F01J    F01K    C01L    C01M    F01N    F01O    F01P
              C01Q    C01R    C01S    C01T    C01U    C01V    C01X    C01Y
              C01Z    C020    F021    C022    C023    C024    H024    C025
              O026    H026    C027    C028    H028    C02A    C02B    H02B
              C02C    H02C    C02D    C02E    C02F    H02F    C02G    C02H
              C02I    H02I    C02J    C02K    H02K    C02L    C02M    C02N
              C02O    C02P    H02P    F02Q    C02R    C02S    H02S    F02T
              C02U    H02U    C02V    H02V    C02X    C02Y    C02Z    H02Z
              C030    H030    F031    C032    H032    F033    F034    C035
              H035    C036    C037    H037    C038    F03A    C03B    H03B
```

```
PLAT802_ALERT_4_G CIF Input Record(s) with more than 80 Characters          1 Info
PLAT860_ALERT_3_G Number of Least-Squares Restraints .............        194 Note
PLAT883_ALERT_1_G Absent Datum for _atom_sites_solution_primary ..     Please Do !
PLAT910_ALERT_3_G Missing FCF Reflection(s) Below Theta(Min)[Deg]=       3.34 Note
                0  0  1,
PLAT912_ALERT_4_G Missing # of FCF Reflections Above STh/L=  0.600        240 Note
PLAT933_ALERT_2_G Number of HKL-OMIT Records in Embedded .res File          1 Note
               13  8  3,
PLAT941_ALERT_3_G Average HKL Measurement Multiplicity ..........         3.6 Low
PLAT969_ALERT_5_G The 'Henn et al.' R-Factor-gap value ..........       3.141 Note
              Predicted wR2: Based on SigI**2  4.59 or SHELX Weight 14.04
PLAT978_ALERT_2_G Number C-C Bonds with Positive Residual Density.          0 Info
```

```
   0 ALERT level A = Most likely a serious problem - resolve or explain
   2 ALERT level B = A potentially serious problem, consider carefully
  24 ALERT level C = Check. Ensure it is not caused by an omission or oversight
  38 ALERT level G = General information/check it is not something unexpected

   5 ALERT type 1 CIF construction/syntax error, inconsistent or missing data
  32 ALERT type 2 Indicator that the structure model may be wrong or deficient
  10 ALERT type 3 Indicator that the structure quality may be low
  14 ALERT type 4 Improvement, methodology, query or suggestion
   3 ALERT type 5 Informative message, check
```

Validation response form

Please find below a validation response form (VRF) that can be filled in and pasted into your CIF.

```
# start Validation Reply Form
_vrf_STRVA01_lcz064
;
PROBLEM: Flack test results are ambiguous.
RESPONSE: ...
;
_vrf_PLAT041_lcz064
;
PROBLEM: Calc. and Reported SumFormula    Strings  Differ     Please Check
RESPONSE: ...
;
_vrf_PLAT042_lcz064
;
PROBLEM: Calc. and Reported MoietyFormula Strings  Differ     Please Check
RESPONSE: ...
;
_vrf_PLAT090_lcz064
;
PROBLEM: Poor Data / Parameter Ratio (Zmax > 18) ........       7.09 Note
RESPONSE: ...
;
_vrf_PLAT213_lcz064
;
PROBLEM: Atom F4              has ADP max/min Ratio .....        3.6 prolat
RESPONSE: ...
;
_vrf_PLAT220_lcz064
```

```
;
PROBLEM: NonSolvent   Resd 1  F   Ueq(max)/Ueq(min) Range        3.2 Ratio
RESPONSE: ...
;
_vrf_PLAT230_lcz064
;
PROBLEM: Hirshfeld Test Diff for    F03A     --C5       .        6.5 s.u.
RESPONSE: ...
;
_vrf_PLAT234_lcz064
;
PROBLEM: Large Hirshfeld Difference F031     --C022     .       0.23 Ang.
RESPONSE: ...
;
_vrf_PLAT241_lcz064
;
PROBLEM: High   'MainMol' Ueq as Compared to Neighbors of       O00U Check
RESPONSE: ...
;
_vrf_PLAT242_lcz064
;
PROBLEM: Low    'MainMol' Ueq as Compared to Neighbors of       C01S Check
RESPONSE: ...
;
_vrf_PLAT369_lcz064
;
PROBLEM: Long   C(sp2)-C(sp2) Bond  C01X     - C02E     .       1.54 Ang.
RESPONSE: ...
;
_vrf_PLAT911_lcz064
;
PROBLEM: Missing FCF Refl Between Thmin & STh/L=    0.600          7 Report
RESPONSE: ...
;
_vrf_PLAT915_lcz064
;
PROBLEM: No Flack x Check Done: Low Friedel Pair Coverage         68 %
RESPONSE: ...
;
_vrf_PLAT971_lcz064
;
PROBLEM: Check Calcd Resid. Dens.  1.35Ang From C02S            1.88 eA-3
RESPONSE: ...
;
_vrf_PLAT977_lcz064
;
PROBLEM: Check Negative Difference Density on H1        .      -0.51 eA-3
RESPONSE: ...
;
# end Validation Reply Form
```

It is advisable to attempt to resolve as many as possible of the alerts in all categories. Often the minor alerts point to easily fixed oversights, errors and omissions in your CIF or refinement strategy, so attention to these fine details can be worthwhile. It is up to the individual to critically assess their own results and, if necessary, seek expert advice.

PLATON version of 17/09/2025; check.def file version of 17/09/2025

---

duplicate check

No duplication found

Datablock lcz064 - ellipsoid plot

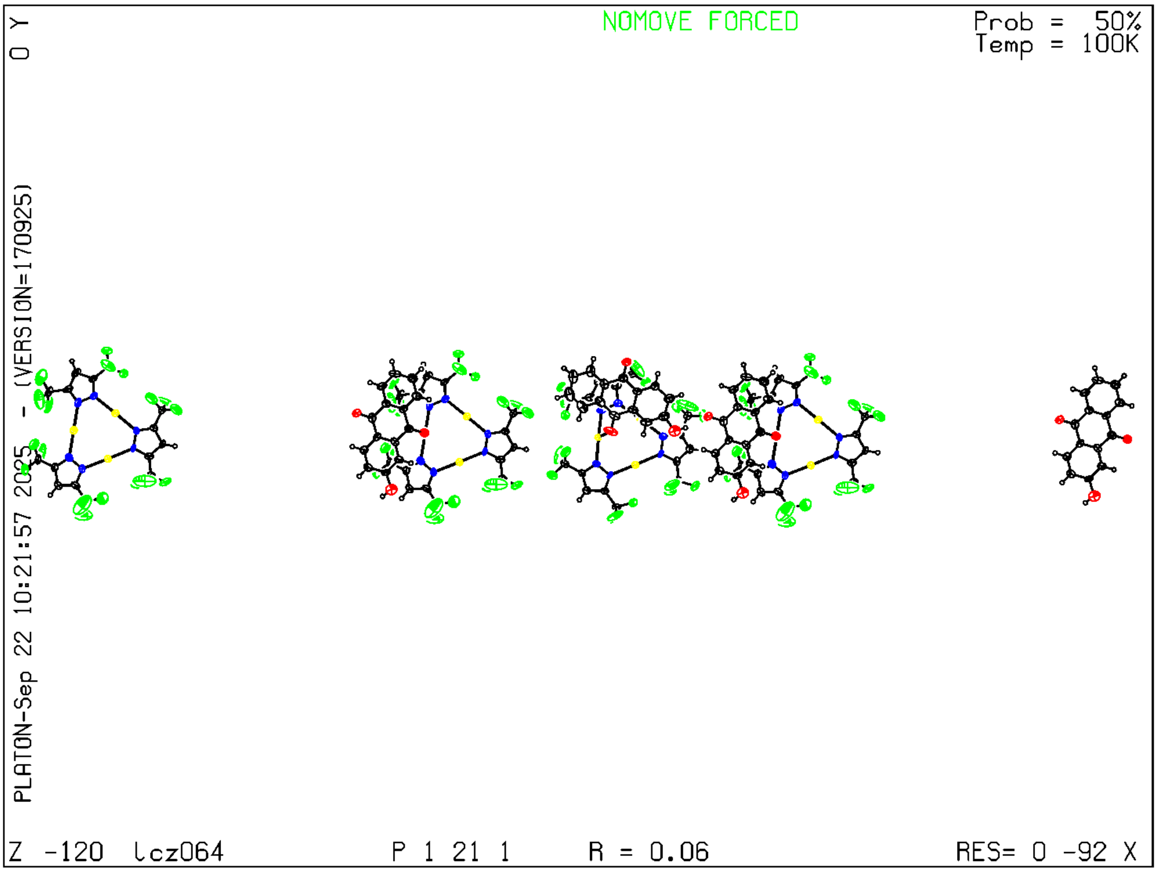

Supplement: Supplementary file 2 — Supporting File 2: anie72462‐sup‐0002‐DataFile.zip. [file ANIE-65-e1218503-s002.zip › supplementary_materials_crystallographic_data submit.zip!/cif_and_checkcif(1-114)/Ag3Pz3_64/Ag3Pz3_64_checkcif.pdf]
